# Evaluation of bioactivity of butternut squash (*Cucurbita moschata* D.) seeds and skin

**DOI:** 10.1002/fsn3.1602

**Published:** 2020-05-14

**Authors:** Haoxin Li

**Affiliations:** ^1^ College of Food Science Guizhou medical university Guizhou China

**Keywords:** ACE inhibition, antioxidant activity, bioactive peptides, butternut squash, waste, α‐amylase inhibition

## Abstract

Butternut squash is greatly consumed in United Kingdom and as by‐products of its processing are produced large amounts of skin and seeds. However, little research has been reported on the antioxidant properties and bioactive peptides from butternut squash seeds and skin. This study focused on assessing the potential of these wastes as sources of beneficial and bioactive compounds. The results indicated that the squash skin phenolic extract showed higher values of antioxidant activity and phenolic content compared with the values of phenolic for the seed material (3.20 mg GAE/g, 1.82 mg GAE/g, respectively). Furthermore, both squash seed protein hydrolysate and skin phenolic extract inhibited α‐amylase activity in a dose‐dependent manner (5–20 mg/ml). Hydrolyzed peptides from squash seeds possess antihypertensive ability (which was significantly different from the control group *p* < .05). Therefore, it can be demonstrated that these squash residues are potentially good sources of bioactive compounds with health benefits.

## Graphical abstract

This study focused on assessing the potential of these wastes as sources of beneficial and bioactive compounds. The results indicated that the squash skin phenolic extract showed higher values of antioxidant activity and phenolic content compared with the values of phenolic for the seed material. It can be demonstrated that these squash residues are potentially good sources of bioactive compounds with health benefits.

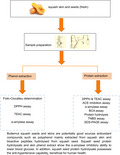

## 1 INTRODUCTION

In recent years, more attention has been focused on the reutilization of food‐processing by‐products and waste. Only a small part of plant or vegetable material is utilized directly for human consumption, whereas a large amount of remaining portion of this material is undervalued, and it may be transformed into fertilizers or feedstuff.

The management of food waste is one of the biggest challenges encountered by many countries. Improper management of food and agricultural waste exerts a severe impact on environmental pollution all over the world, in light of the biodegradable characteristics of food product with high content of water which produced bad odor and abundant leachate (Cheng & Hu, 2010) and emission of greenhouse gases (i.e., methane) in landfills (Liu et al., 2012). Specifically, approximately 20 megatonnes of greenhouse gas emissions (CO_2_ equivalents) a year and many billions of tonnes of water, the majority of which is associated with the production, transport, and packaging of food waste rather than its disposal. According to the waste prevention program for England, food waste was one of the priority waste streams. In addition, from an economic perspective, the great waste of food ingredients during processing and packaging waste in the UK food supply chain is evaluated at GBP 6.9 billion a year, which is equivalent to more than 10% of gross value added (Parry et al., 2015).

However, in fact the remaining portion of the food‐processing by‐products and wastes may be converted into fertilizers, while it should have an important contribution to food resources or industrial products considering the abundant nutritions contained in these residues (Kamel, Deman, & Blackman, 1982).

Lately, more attention has been gained on the squash owing to the nutritional value of the seeds and skin that are abundant in health beneficial compounds like vitamins, polysaccharides, carotene, mineral salts, and others (Fu, Shi, & Li, 2006; Fu, Tian, Cai, Liu, & Li, 2007; Zhemerichkin & Ptitchkina, 1995). Specifically, a study showed that squashes contain pectin in their cell walls, a polysaccharide that typically contains chains of d‐galacturonic acids. These components in the squash have the anti‐inflammatory properties as well as properties that assist in warding off diabetes and modulating insulin levels (Fu et al., 2006). Evidence from a study indicated butternut squash is beneficial in combating macular degeneration, heart health, and immune function due to the quite high level of carotenoids which are able to convert to vitamin A in the body; particularly, beta‐carotene is beneficial in preventing cancer cell growth by turning on a gene in the body that can encourage cell communication. Moreover, the high fiber content of squash also played a positive role in colorectal carcinogenesis (Kim, 2000). In addition, there are lot of studies that indicate the nutritional value fatty acids from squash seed oils and melon skin (Murkovic, Hillebrand, Winkler, & Pfannhauser, 1996), such as polyunsaturated omega‐3 fatty acids known for their anti‐inflammatory properties and their health benefits related to cardiovascular health.

In reality, after removing the flesh from squash there remains a large amount of seeds and skin as waste products. It is reported that these squash waste residues are excellent sources of protein and phenolic compounds. However, there is little in literature on bioactive peptides and phenolic antioxidant activity in squash. The biopeptides can be regarded as biological active amino acid chain joined together, in terms of the formation of these biologically active peptides; after enzymatic hydrolysis, previously inactive amino acid sequence is liberated and exerted advantageous properties. In addition, bioactive peptides have health benefits functioning as antihypertensives, antioxidant, immunomodulating, anticarcinogens, antimicrobials, and others. Considerable evidence shows that the increased oxidative damage is linked with the age‐related degenerative diseases (Jessica et al., 2005). As regard to the phenolic, it plays a key role as antioxidants that may exert positive effects against such conditions due to the presence of hydroxyl substituents and their aromatic structure, which enables them to scavenge free radicals. In light, some antioxidants cannot be synthesized by the human body, while phenolic which mainly presents in squash has an important role in protection against oxidative stress and can be obtained and digested through diet. These phenolic compounds are significant to total water‐soluble antioxidant activity (Tadmor, Paris, Meir, Schaffer, & Lewinsohn, 2005), and it may protect biomolecules from oxidative damage and thus correlate with reduced risks of degenerative diseases, cardiovascular disease, and certain cancer (Chen et al., 2008).

Little research has been performed on the antioxidant properties of squash seeds and skin, the bioactive peptides, and the way optimizing utilization of these wastes from squash. This study mainly focuses on elucidating the potential of squash waste following processing and to investigate the health benefits and nutritional value of these compounds from waste squash seeds and squash skin, evaluating the presence of antioxidant activity and bioactive peptides. Therefore, it could contribute to maximizing the available potential of these waste residues and result in transforming them into co‐products with added value.

## 2 MATERIALS AND METHODS

### 2.1 Chemicals

All chemicals, solvents, and reagents were of analytical grade. Water was ultrapure.

### 2.2 Plant material

The samples of butternut squash (Cucurbita moschata) seeds and skin used in the study were purchased from a large supermarket company (Tesco stores Ltd.) localized in Leeds, West Yorkshire.

**FIGURE 1:**
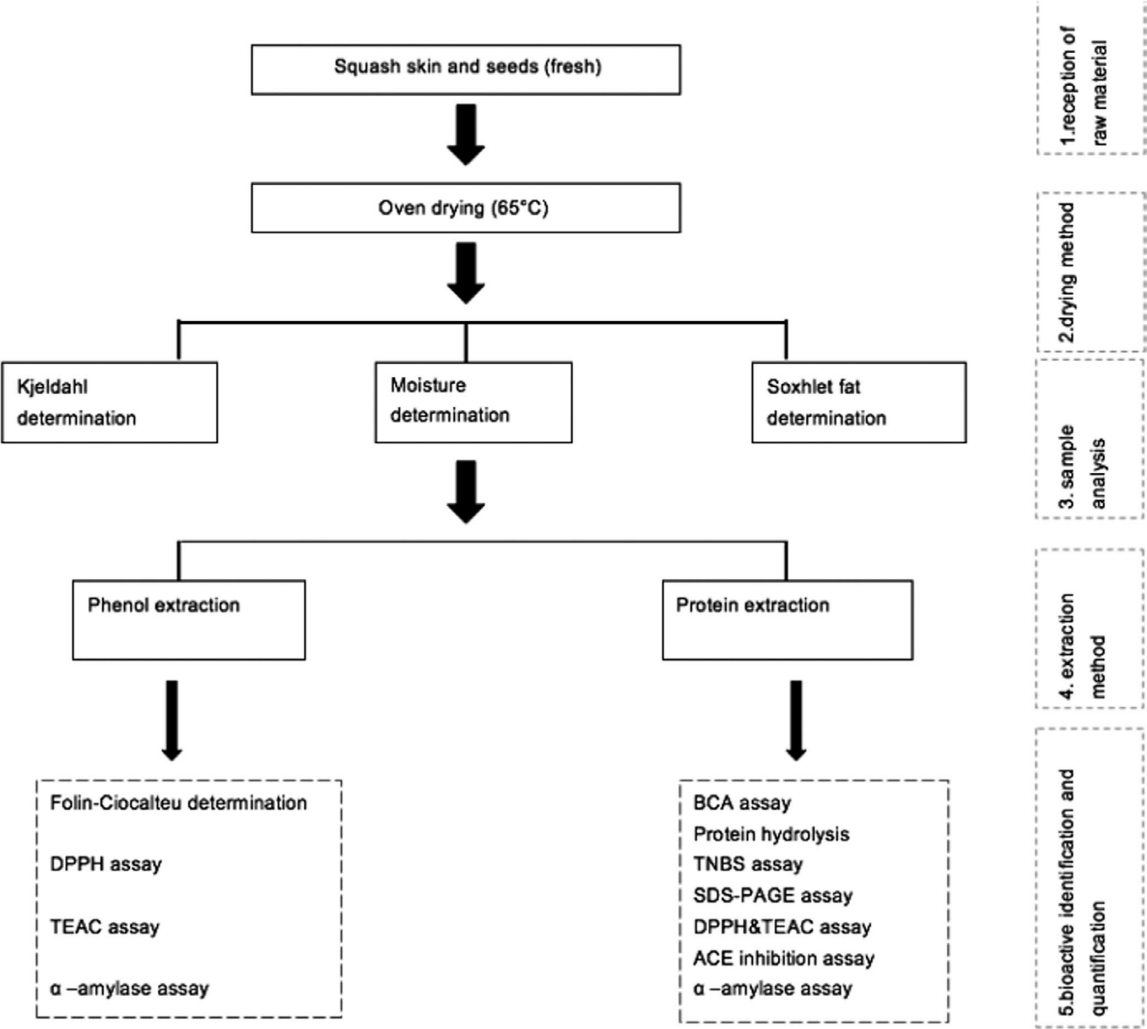
Processing scheme of the squash residues from the supermarket

Immediately transported to the laboratory, all samples were refrigerated at 4°C until analysis. Uniform size and color butternut squash were selected and then were washed thoroughly with tap water and peeled. The seeds were removed using a spatula. Fresh squash seeds and skin were dried using air‐drying in an oven (65°C) overnight. The samples were then powdered using Jaipan 750 Watts mixer grinder machine (Jaipan Industries Ltd.) (Figure 1).

### 2.3 Determination of crude protein content in seed and skin of butternut squash

Total nitrogen was estimated using the Kjeldahl method as described by Kjeldahl in 1883. Two g of samples was initially digested using 25 ml of 1 M H_2_SO_4_ (7664‐93‐9) with the addition of a Kjeldahl catalyst tablet (Gerhardt lab supplies Ltd.) using a turbotherm digestion system purchased from C. Gerhardt UK Ltd for 3 hr at the condition of program 100. The resultant digestion was distilled and titrated the ammonia contained in the 4% (w/v) boric acid solution (10043‐35‐3) with 0.25 M sulfuric acid (7664‐93‐9). The amount of total protein was calculated as percentage % (titration volume cm^3^ × 0.0070 g/cm^3^ × 6.25/2 g).

### 2.4 Determination of fat content in seed and skin of butternut squash

The fat content from ground samples was determined using solvent in a Soxhlet apparatus. It contained 2 stages, separation and extraction. Approximately 12.0 g ground butternut squash skin or 8.0 g seed was weighed, then placed in the Soxhlet extraction thimble, inserted pieces of cotton on the top of the thimble (in case of leakage), extracted with 150 ml petroleum ether (64742‐49‐0, VWR, France) for 24 hr, and then went though the clean‐up procedure. Finally, the fat content of samples was evaluated. The results were expressed as % of fat per sample mass.

### 2.5 Extraction of polyphenol

In order to evaluate the phenolic antioxidant activity, the phenolic should be extracted from the butternut squash skin samples. The subsamples of the fresh material (3 × 500 mg) were extracted with 10 ml of 70%methanol (67‐56‐1) at 70°C for 30 min in 10‐ml screw‐top tubes, using a vortex mixer every 5 min to optimize extraction. The samples were centrifuged (17,000 *g*, 4°C, 20 min), and subsamples of the supernatant (3 × 100μl) were prepared for assays and stored in the ultra‐low temperature freezer (Panasonic Healthcare Co., Ltd.) at −80°C.

### 2.6 Extraction of protein

This assay was performed to isolate the protein from the butternut squash seed sample according to a previously described procedure (He, Yang, & Zhang, 2014), with some modifications. The properties of these purified protein can be evaluated through following assays. 100 g of dried squash seed and skin flour was stirred for 1 hr at room temperature in 1,000 ml distilled water. The resultant slurry was centrifuged at 2,200 *g* for 10 min, and the supernatant was collected. The leftover was extracted again with 500 ml of distilled water for 1 hr and separated with centrifugation as mentioned above. Then, supernatants were pooled and the pH was adjusted with 1.0 mol/L HCl (7647‐01‐0) to pH 4 using Hanna HI 208 pH meter (Hanna instruments Inc. Romania), where most of the protein was precipitated. Then centrifuged at 4,000 *g* for 15 min and subsamples of the supernatant were freeze‐dried using Christ freeze dryer machine (MechaTech Systems Ltd.) and stored in the freezer at −30°C.

### 2.7 Determination of BCA Assay

In order to determine the isolated protein fractions content, the bicinchoninic acid assay was adopted as it can be applicable to any substrate and without interference on its surface properties (Cieplik et al., 2013). The protein content of the isolated samples was determined using the BCA method (Pierce™ BCA Protein Assay Kit' Prod #23227; Thermo Scientific). A 2.0 mg/ml albumin protein stock solution was diluted to make a set of standard solution with the protein concentration in a range of 0 to 2,000 µg/ml. One mg of isolated sample was dissolved in 1 ml of distilled water. A 25 µl of each standard or sample solution was mixed with 200 µl working reagent (50 parts of Reagent A with 1 part of Reagent B, 50:1), on a 96‐well plate, mixed thoroughly on a plate shaker for 30 s and incubated at 37°C for 30 min. The absorbance was then measured at 562 nm using a Spark 10 M Tecan Spectrophotometer. The protein content of isolated samples was expressed as μg/ml of triplicate experiments.

### 2.8 Pepsin hydrolysis

In order to hydrolyze the protein extract samples, the samples were diluted with simulated gastric fluid (7732‐18‐5) (SGF, containing 0.034 M NaCl 7647‐14‐5, pH 1.2). The final concentration was 9.0 mg/ml. All samples were stirred using a magnetic stirrer in a 37°C shaking GLS Aqua 12 Plus water bath (Grant instruments Ltd.). Previous assay performed by He et al. (2014) found that a fresh pepsin solution (20 mg/ml in SGF solution) stirred with a magnetic stirrer for 10 min before it was centrifuged using a MiniSpin centrifuge (Eppendorf) at 10,000 *g* for 3 min to remove the insoluble substances and then added to these solutions. The final concentration of pepsin was 2 mg/ml. One ml of 10 mg/ml butternut squash seed protein extracts was withdrawn, and digestion was terminated by adding 0.2 M Na_2_CO_3_ (497‐19‐8) after 0, 10, 20, 30, 60, 90, and 120 min. During the hydrolysis process, samples of the protein hydrolysates were taken periodically. The pH was adjusted to 2 by the addition of 1 mol/L NaOH or 1 mol/L HCl using Hanna HI 208 pH meter (Hanna instruments Inc. Romania) which is the optimal pH for the protein hydrolysis (Xiu‐Ting, Xiao‐Quan, & Jin‐bo, 2014). A 100% hydrolyzed squash seed protein extract (10 mg/ml) was prepared using 6 M HCl (7647‐01‐0) and heated for 24 hr at 100°C using a QBT1 digital block heater (Grant Instrument Cambridge Ltd.).

### 2.9 Determination of degree of hydrolysis

The degree of hydrolysis (DH) at each stage of the digestion evaluated by determination the amount of freed alpha amino groups in the squash seed and skin protein hydrolysate was carried out by TNBS colorimetric method. Previous study performed by Spellman et al. (2003) proved that TNBS method was an excellent method for quantifying DH, regardless of the type of enzyme activity used for hydrolysis. 1 M TNBS 2,4,6‐trinitrobenzene sulfonic acid (2508‐19‐2) is a highly sensitive chemical used to measure the amount of free amino groups. The reaction of TNBS with primary amines generated a highly chromogenic product that can be readily measured at 335 nm. One mg/ml of protein solutions was prepared in reaction buffer diluted 50 times (0.1 M sodium Bicarbonate 144‐55‐8 pH 8.5). Immediately prior to assaying, prepare a working solution of 0.1% (w/v) TNBS in reaction buffer. Different concentrations of standard solution were made from L‐leucine (61‐90‐5) varies from 0 to 0.5 μmol/ml. Then, adding 62.5 μl working solution of TNBS to 125 μl each sample (1 mg/ml) and standards and mixed well. The reaction was incubated (MIR‐262 incubator SANYO Electric Co., Ltd.) at 37°C for 2 hr; then, 62.5 μl 10% SDS and 31 μl 1 M HCl were added to stop the reaction. The absorbance was measured at 335 nm. The hydrolysis degree was defined as the % ratio of the number of peptide bonds broken (h) to the total number of bonds per unit weight (htot) and was calculated as: DH = (h/htot) × 100%. The experiment was performed in triplicate.

### 2.10 Determination of protein content using gel electrophoresis

SDS‐PAGE was performed on a discontinuous buffer system at 4%–15% Mini‐PROTEAN TGX precast gel (pH 6.8) (Bio‐Rad Laboratories, Inc.) and separating gel (pH 8.8) using gel electrophoresis apparatus. Nine volumes of 100% ethanol were added to 1 volume of protein solution, mixed, and incubated at −20°C for 60 min. Then, samples were centrifuged in microcentrifuge at 15,000 *g* for 15 min. Samples (20 µl 10 mg/ml protein for each) and 5 µl loading dye (Bio‐Rad Laboratories. Inc.) were loaded on gel. Electrophoresis was performed at 120 V with BIO‐RAD basic Electrophoresis Power Supply (Bio‐Rad Laboratories. Inc.) in the separating gel until the tracking dye reached the bottom of the gel. After electrophoresis, the gel was stained with Safe blue (6104‐58‐1) (0.25% Blue‐G250) in ethanol (ethanol/staining solution = 1:9, v/v).

### 2.11 Determination of total phenolic content in seeds and skin of butternut squash

Total phenolic content from squash residues was determined by the Folin–Ciocalteu method (Slinkard & Singleton 1997) by using GA (gallic acid) as a standard for the calibration curve. A 20 µl of extract sample and standards was incubated with 100 µl Folin–Ciocalteu's reagent and left to stand at room temperature for 5 min, and then, 80 µl of 7.5% (w/v) Na_2_CO_3_ (497‐19‐8) was added and the solution incubated at room temperature for 2 hr. Gallic acid 3, 4, 5‐trihydroxybenzoic acid (149‐91‐7) was used as a standard ranged from 0 to 100 µg/ml. The absorbance was read at 760 nm using Spark 10 M Tecan Spectrophotometer. The results were expressed as gallic acid equivalent mg GAE/g. Data were expressed as means ± *SD* of triplicate experiments and determined by a calibration curve (*y* = 0.0049*x* + 0.0196, *R* ^2^ = .99656).

### 2.12 Determination of antioxidant activity

#### 2.12.1 Determination of radical scavenging activity of seeds and skin of butternut squash

The antioxidant activity (AA) was evaluated using DPPH 2.2‐difenil‐1‐picrilhidrazil radical (84077‐81‐6) method. The free radical DPPH• is degenerated to the corresponding hydrazine when reacted with hydrogen donors, and the DPPH• signal intensity is inversely correlated with the antioxidant concentration and reaction time (Peschel et al., 2006). Different concentrations (0.0 to 1 mM) of trolox stock solution were prepared, diluting the stock solution in methanol 60% (methanol: water, v/v). Twenty µl 5 mg/ml methanolic extracts (including components of squash seed and skin) and standards were mixed with 200 μl of a methanolic solution containing DPPH• radicals (0.1 mM in methanol 100%). This mixture was agitated and kept in dark up to 1 hr, in order to obtain stable values of absorbance. Finally, this free radical scavenging ability of DPPH• was evaluated by this decolouration assay where the decrease in absorbance at 517 nm produced by the addition of the antioxidant to DPPH• in methanol using Spark 10 M Tecan Spectrophotometer. Data were expressed as means ± *SD* of triplicate experiments. The antioxidant activity of each extract was expressed as (%) DPPH scavenging activity, accordingly to the equation:

$$\left(\%\right)\;\text{scavenging activity}=\left[\left({\text{Abs}}_{\text{blank}}-{\text{Abs}}_{\text{sample}}\right)/{\text{Abs}}_{\text{blank}}\right]\times 100$$

#### 2.12.2 Determination of the Trolox Equivalent Antioxidant Capacity (TEAC) value

TEAC assays based on the antioxidant ability of a compound to scavenge the blue‐green colored stable ABTS radical (ABTS*) in 5 min. The ABTS* solution was prepared through the reaction between 7 mM ABTS and 2.45 mM potassium sulfate and dilute to give an absorbance of about 0.6 at 734 nm. This solution was stored in the dark at least 12 hr before use. The concentrated trolox solution (6‐hydroxy‐2,5,7,8‐tetramethylchromane‐2‐carboxylic acid (53188‐07‐1) was diluted with phosphate‐buffered saline (PBS) for standard curve (0–100 µg/ml in PBS reaction buffer). The squash samples including hydrolyzed protein from squash seed and extracted polyphenol from squash skin (45 μg/ml and 80 μg/ml, respectively) were diluted 20 times using PBS. The trolox standards and samples (5 µl) were added to 250 µl ABTS solution. The decrease in absorbance caused by the antioxidant compounds measured after 5 min at room temperature. The experiment was performed in triplicate. The TEAC value can be expressed in mM of trolox equivalents.

### 2.13 Determination of ACE inhibitory activity

The aim of the ACE inhibition assay was to determine the antihypertensive effects of enzymatic protein hydrolysate from squash residues. Seed protein isolate was digested by the pepsin to simulate the gastrointestinal digestion in human beings. The ACE inhibition activity of squash seed peptide fractions was evaluated as previously reported with modifications (Udenigwe, Lin, Hou, & Aluko, 2009). Briefly, 50 µl of 2 mM FAPGG (64967‐39‐1) (dissolved in 50 mM Tris–HCl buffer containing 0.3 M NaCl, pH 7.5) was mixed with 10 μl ACE (1 U/ml) (9035‐69‐2) and 40 μl sample (1 mg/ml) in 50 mM Tris–HCl buffer. The rate of decrease in absorbance at 340 nm was recorded every 5 min for 30 min at room temperature. As regard to the control, the Tris–HCl buffer was used instead of squash peptide fractions.

### 2.14 Determination of α‐amylase inhibitory activity

This assay was used to assess the samples α‐amylase inhibitory properties. The squash sample extracts (1 mg/ml) diluted by 10 degrees (50 μl) were mixed with 500 μl of 0.02 mol/L sodium phosphate buffer (pH 6.9 with 0.006 mol/L NaCl) and 100 μl of 0.5 mg/ml porcine pancreatic α‐amylase (9000‐90‐2) (Sigma Aldrich Co., Ltd. #product A3176) and incubated at room temperature for 10 min. Then, 500 μl of 1% starch solution (9005‐25‐8) was added to the reaction mixture. The reaction mixture was incubated for 10 min at room temperature, and 1.0 ml of DNSA dinitrosalicylic acid (609‐99‐4) was added. The reaction was ceased by incubating in a boiling water bath for 5 min and cooled to room temperature. Then, diluted by adding 10 ml of distilled water, and absorbance was measured at 540 nm using Multiskan™ FC Microplate Photometer (Thermo Fisher Scientific Instruments Co., Ltd.). The reference sample included all other reagents mentioned and the enzyme except the test sample. Assays were performed with a range of squash phenol and protein contents.

The α‐amylase inhibitory activity was expressed as percentage inhibition (Worthington, Weddell, & Neilson, 1993).

$$\text{Inhibition}\;\left(\%\right)=\left(\frac{{\text{Abs}}_{\text{ref}}-{\text{Abs}}_{\text{sample}}}{{\text{Abs}}_{\text{ref}}}\right)\times 100$$

$${\text{Abs}}_{\text{ref}}=\text{Absorbance of the reference}$$

$${\text{Abs}}_{\text{sample}}=\text{Absorbance of the test samples}$$

### 2.15 Statistical analysis

All experiments were performed in triplicate, and data were presented as the mean ± *SEM* (standard error of the mean). The differences between the mean values were evaluated using Student's *t* test, and differences were considered to be significant if *p* < .05.

## 3 RESULTS

### 3.1 Determination of fat, protein, and moisture content in seed and skin of butternut squash

Values of both protein and fat content for squash seed (32.38% and 14.31%, respectively) were higher than those of squash skin (14.06% and 0.63%, respectively). The skin samples contained very high moisture, and they each have a moisture content over 80%, while the seed sample moisture content accounted for only a small proportion around 25%. The fat level from butternut squash skin samples had the largest coefficients of variation, which can be ascribed to the tiny fat content contained in the sample, and the error range was liable to be higher than other chemical composition levels (Table 1).

**TABLE 1:** Average chemical composition of butternut squash skin and seed samples (% w/w) (*n* = 3)^a^

Chemical component	Squash skin		Squash seed	
	av	SE	av	SE
Protein	14.06 a	0.42	32.38	0.68
Fat	0.63 a	0.28	14.31	0.22
Moisture	80.75	0.23	28.74 a	0.16

^a^ A value that is significantly greater (*p* < .05) than its paired value is denoted with an “a.” Paired results with no letter have no significant difference.

After the protein extraction procedure, the squash seed isolated protein content (566.31 μg/ml) was approximately fivefold higher than squash skin isolated protein content (123.46 μg/ml) based on the result of BCA test. In addition, the isolated protein concentration determined by the BCA assay is positively correlated with result assessed by the Kjeldahl assay (the crude protein content of squash seed is approximately twofold higher than the squash skin sample 14.06% and 32.38%, respectively). There existed significant difference between the isolated protein levels of two components of squash (*p* = .032 < .05). As regard to the isolated protein yield of extracts, the yield value of squash seed was fivefold higher than that of squash skin (57.18% and 11.39%, respectively). These data are the first to be reported.

### 3.2 Degree of hydrolysis

**FIGURE 2:**
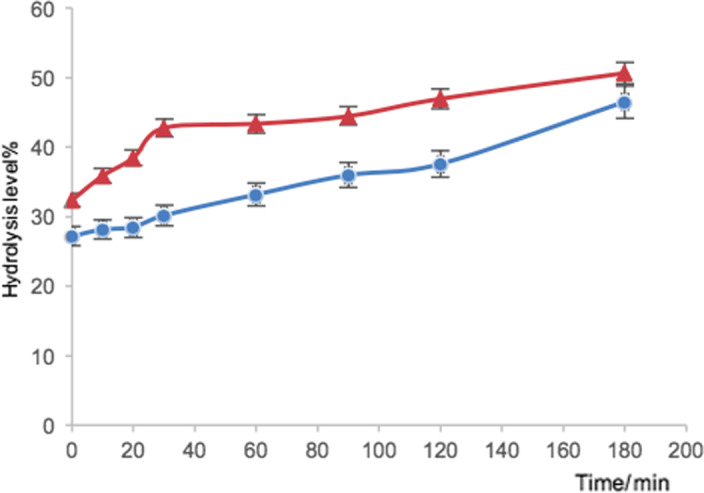
The degree of hydrolysis DH (%) values obtained by the TNBS method for the hydrolysis of squash seed protein extract (∆) and squash skin protein extract (○) within 180 min (*n* = 3). Error bars indicate standard deviation

Two different squash residues (skin and seed protein extract) were compared in this assay. Overall, the degree of hydrolysis (DH) of squash seed protein extract was higher than the skin protein extract within the 180‐min period using TNBS assay. It can be clearly shown that the degree of hydrolysis of seed protein extract (32.4%) was higher than the squash skin protein extract (27.2%) at beginning (0 min), and with the time increasing, the hydrolysis level was elevated simultaneously. As regard to the hydrolysis rate, the DH of squash seed sample increased rapidly at first phase (0–30 min), and with the time increased, the reaction rate slowed down, whereas the DH value of squash skin sample increased consistently within the 180‐min period. The most extensive hydrolysis occurred where the DH reached approximately 50% (50.7% for squash seed and 46.5% for squash skin, respectively) after 180 min. A possible explanation for the different hydrolysis levels could be related to the amount of protein contained in samples. As the BCA and Kjeldahl assay results shown above, the squash seed samples contained much higher crude and isolated protein content than squash skin. In addition, the hydrolysis level of both squash seed and skin protein hydrolysate did not reach plateau thus required digest for longer to determine when hydrolysis finishes in future research (Figure 2).

### 3.3 Molecular mass of protein hydrolysates

**FIGURE 3:**
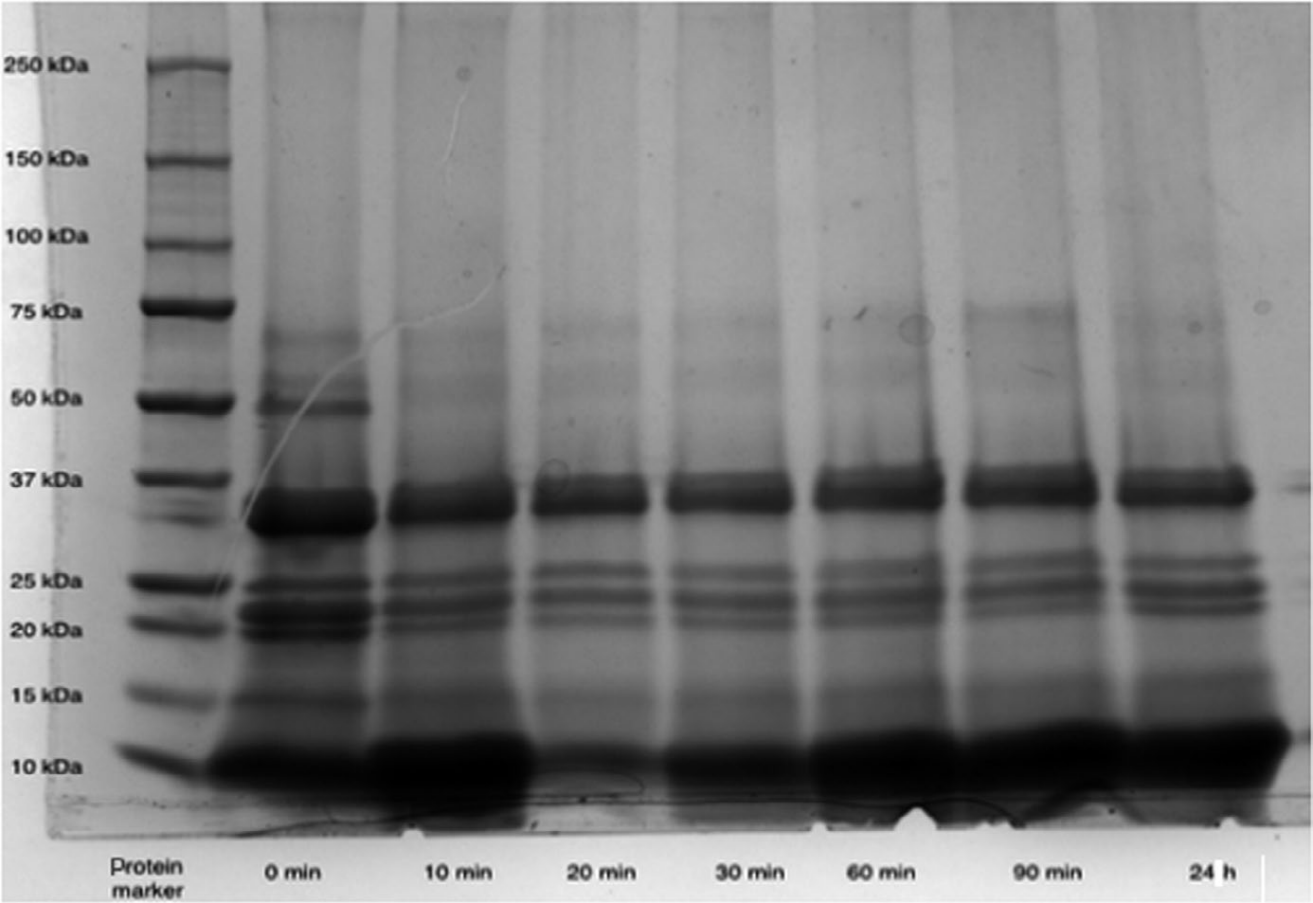
SDS‐PAGE patterns of butternut squash seed protein hydrolysate. Lane 1 shows molecular mass markers from top downward (250–10 kDa). Lanes 2 to 8 indicate electrophoretic mobility of squash seed extract hydrolyzed from 0 min to 24 hr

At the beginning, these protein hydrolysates degraded with the enzymatic function of pepsin (50 kDa). The pepsin can be observed in each band of squash seed protein hydrolysate samples in which molecular weight is approximately 34 kDa. With the hydrolysis time increased, the squash seed protein extract began to degrade to smaller fragments (peptides) as the figure showed between 0‐ and 10‐min digestion. Finally, bands of squash seed protein hydrolysate with a molecular mass below 15 kDa were observed due to protein degradation (Figure 3).

### 3.4 Phenolic content

The total phenolic content was determined using Folin–Ciocalteu method. Compared with the efficiency between two different extraction solvents, higher values of the extraction with 70% methanol (1.82 ± 0.05 mg GAE/g for squash seed and 3.20 ± 0.03 mg GAE/g for squash skin) were detected, while the mixed solvent methanol: water: formic acid (50:48:2) showed the lower values (1.35 ± 0.01 and 2.79 ± 0.12 mg GAE/g) for squash seed and skin samples, respectively. The skin samples indicated higher values (2.79 and 3.20 mg GAE/g) of total phenolic content than the seeds samples (1.35 and 1.82 mg GAE/g). On the other hand, there was significant difference between the two different extraction methods as regard to the squash seed samples (*p* < .05), while no significant difference existed between these extraction method regarding to the squash skin samples (*p* > .05). A possible explanation could be the phenol content is too low to be distinguished between different extraction solvents.

### 3.5 Antioxidant activity

Owing to the existence of many reaction characteristics and mechanisms, a single antioxidant experiment will not accurately reflect the overall antioxidant activity. Thus, the use of different antioxidant assays assists in identifying variations in the response of the compounds extracted from squash samples. For this reason, antioxidant activity was measured by 2 methods (DPPH and TEAC assays). Two different antioxidant assays were performed to indicate different aspects of the antioxidant capacity of extracts (phenol and protein extract) from butternut squash.

The DPPH assay measures the ability of antioxidants to scavenge the DPPH radical (DPPH•), whereas the scavenging of the ABTS radical (ABTS•) converting it into a colorless product.

The butternut squash skin samples presented lower IC_50_ values (12.42 ± 5.23) of antioxidant activity than the seeds samples (106.78 ± 14.96). As regards to the result of TEAC assay, Table 2 shows the TEAC IC_50_ value of the squash skin phenol extract sample (21.28 ± 0.55) which was ninefold lower than the seed protein extract sample (186.45 ± 0.64). There existed significant difference between the antioxidant activities of squash skin and seed phenol extracts measured by DPPH and TEAC assay. (*p* < .05).

**TABLE 2:** The total phenol content, DPPH IC_50_ value, and TEAC IC_50_ value in butternut squash seed and skin samples

Components of sample	DPPH IC_50_ (µg/ml)	TEAC IC_50_ (µg/ml)	Phenol content (µg/ml)
Squash skin	12.42 ± 5.23	21.28 ± 0.55	79.97 ± 0.86
Squash seed	106.78 ± 14.96	186.45 ± 0.64	45.45 ± 1.36

Note

The results are presented as mean ± standard deviation (*n* = 3), *p* < .05 between phenol content of squash polyphenol extracts and antioxidant activities measured by DPPH&TEAC assay.

### 3.6 α‐amylase

**FIGURE 4:**
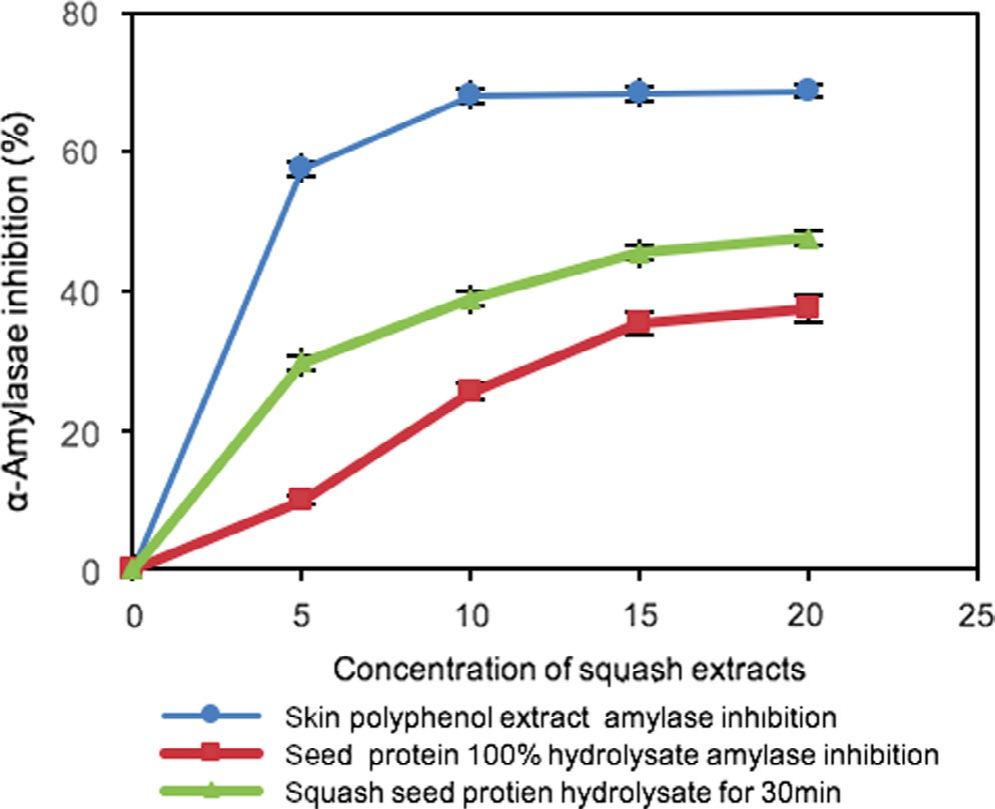
α‐Amylase inhibitory activities of butternut squash residues (seed and skin extract). Values are means of duplicate determinations

The result revealed that both squash seed protein hydrolysate and skin phenolic extract inhibited α‐amylase in a dose‐dependent manner (5–20 mg/ml). There existed a significant difference between the squash seed protein hydrolysate and skin polyphenol extract in light of the *p*‐value <.05. However, there was no significant difference between squash seed protein hydrolysate (*p* > .05). In general, the squash skin phenol extract exhibited the highest inhibitory activity with the IC_50_ value (4.34 mg/ml). The squash seed protein hydrolysate hydrolyzed by pepsin for 30min gave the second highest activity, and the 100% hydrolyzed squash seed peptides presented the lowest α‐amylase inhibitory activity. This can be ascribed to the function of 6 M HCl hydrolysis heated for 24 hr, and the peptides were thoroughly broken down and inactivated under high temperature; thus, the 100% hydrolyzed samples had no bioactive peptides to react with this enzyme; thus, the inhibition activity was lower than another protein hydrolysate for 30‐min digestion (Figure 4).

### 3.7 ACE assay

**FIGURE 5:**
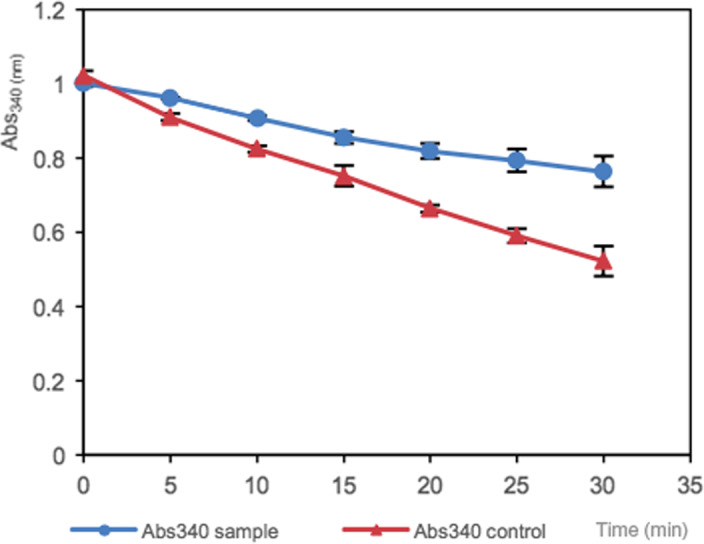
The ACE inhibitory activities in the absence and presence of butternut squash seed peptide fractions. All the results are means of triplicate (*p* < .05)

The squash seed protein hydrolysates showed ACE inhibitory activities owing to the lower decreasing rate compared with the control (−0.0164 and −0.0082, respectively). After 30 min, the ACE inhibition value reached to 31.5% ± 0.04%. There existed extremely significant difference between the ACE inhibition value of sample and control within 30 min (*p* < .05), which demonstrated that after the pepsin treatment of squash seed protein released bioactive peptides from the native protein structure, and these peptides possessed the ACE inhibitory activity (Figure 5).

## 4 DISCUSSION

The phenolic content, isolated protein concentration, antioxidant properties, α‐amylase, ACE inhibitory properties of the peptides, and polyphenol extracts of squash seed and skin were evaluated.

At the beginning of the study, the crude protein, fat, and moisture content were evaluated. Compared with a previous study, Hui‐Yu, Sue, Dee, and K.D. M. (2004) showed 47.85% moisture contents referring to squash seed samples. A possible explanation for this could be the difference in squash variety and storage conditions. After the preliminary analysis of the squash by‐product samples, these squash skin and seed samples were hydrolyzed by pepsin, and the squash seed protein hydrolysate revealed higher hydrolysis level than the squash skin within 180 min. Compared with the previous study (Xiu‐ting et al., 2014) on protein hydrolysis determination on fresh fish (anchovy and cod, respectively), the DH value (around 70%) was much higher than the squash seed protein hydrolysate. This difference may be attributed to several factors including the pH, temperature, and digested efficiency of enzyme and its related concentration, also sequences of protein which will be digested at different cleavage sites due to the amino acid sequence. According to the result of SDS‐PAGE gel assay, due to the protein degradation, squash seed protein hydrolysate finally degraded with a molecular mass below 15 kDa, and these components may be potentially α‐moschin and β‐moschin corresponding to a molecular mass of 12 kDa according to Ng, Parkash, Tso (2002) research on characterization of moschins with translation‐inhibiting activity from brown pumpkin (Cucurbita moschata) seed.

As regard to the phenol content determination, similar trends have been reported for squash samples purchased in the north of Portugal (Saavedra et al., 2015). The squash skin samples presented higher values (4.69 ± 0.82 mg GAE/g) of total phenolic content than the seeds samples (0.95 ± 0.10 mg GAE/g). Another study (Xanthopoulou, Nomikos, Fragopoulou, & Antonopoulou, 2009) showed much lower levels of total phenolics found in pumpkin oilseeds (0.00005–0.011 mg GAE/g). It may be ascribed to the difference between different varieties and the growing environment. In addition, according to Aliki, Ganopoulos, Kalivas, & Nianiou (2015), Peschel et al. (2006), and Turkmen, Sari, and Velioglu (2005) studies used to measure the polyphenol content and antioxidant capacity have used various solvents for extraction with methanol in waste fruits and vegetables having the highest extraction efficiency compared with other solvents such as ethanol and dichloromethane. The phenolic composition of fruits is influenced by several factors such as environmental and storage conditions and genetic variation (Gliszczynska‐Swiglo & Tyrakowska., 2003).

Compared the antioxidant activity with other studies, a recent report (Saavedra et al., 2015) on the antioxidant activity (DPPH) of pumpkin by‐products revealed similar results, and the oven‐dried (65°C) pumpkin skin samples indicated higher levels of antioxidant activity than the seeds samples. On the other hand, compared the TEAC IC_50_ results between squash skin and seed phenol extract, the squash skin phenol extract presented lower 50% inhibitory concentration(IC_50_) (21.28 ± 0.55 µg/ml) which means the antioxidant activity presented higher levels. This result is consistent with DPPH assay. According to studies (Scalzo, Politi, Pellegrini, Mezzetti, & Battino, 2005 and Krishna, Attri, & Kumar, 2014), the antioxidant potential of the analyzed sample is strongly influenced by genotype, heat treatment, and processing effects. In addition, the total phenolic content of squash skin was positively correlated with the TEAC antioxidant activity (*r* ^2^ = .980; *p* < .05). This correlation between bioactive polyphenols and the antioxidant activity of extracts suggests that phenolic compounds may be responsible for the antioxidant capacity in TEAC assay which is consistent with Vison, Hao, Su, and Zubik (1998) and Wang & Lin (2000) studies. These studies demonstrate the antioxidant activity increased with the higher phenol content in the sample extracts.

α‐Amylase is considered to be an excellent test to determine whether the squash seed and skin extract samples possess the ability to slow down the breakdown of starch in the intestinal tract resulting in lower blood glucose levels. It is involved in the dietary starch digestion, with the function of α‐amylase, the starch was broken down into disaccharides and then glucose which can be rapidly absorbed by the blood circulation (Oboh, Akinyemi, Ademiluyi, & Bello, 2010). The trend is consistent with those of the degree of protein hydrolysis, which demonstrates that it is the activity of peptides present in the hydrolysates that are mainly responsible for the inhibition of α‐amylase activity (Chatchaporn & Jian, 2016). In addition, the α‐amylase inhibitory action could be also attributed to the phenolics present in squash skin.

In this assay, there was no IC_50_, a possible explanation of this could be the reaction time was not enough to evaluate the actual inhibitory activity. Compared with a previous study (Chibuike, Yin‐Shiou, Wen‐Chi, & Rotimi, 2009) on kinetics of the inhibition of ACE enzyme by flaxseed protein hydrolysate fractions, the ACE inhibition efficiency is much higher than this assay, which showed the ACE inhibitory activity of flaxseed protein hydrolyzed by thermolysin (61% at 0.05 mg protein/ml). According to the study (Chibuike et al., 2009), the ACE inhibitory activity was based on two major influencing factors, the type of protease to digest and the active peptide sequences liberated.

## 5 CONCLUSIONS AND PERSPECTIVES

The results of this study demonstrate these residues from food processing including butternut squash seeds and skins are potentially good sources antioxidant compounds such as polyphenol mainly extracted from squash skin and bioactive peptides hydrolyzed from squash seed. In addition, both squash seed protein hydrolysate and skin phenol extract show the α‐amylase inhibitory ability to lower blood glucose. In addition, squash seed protein hydrolysate possesses the antihypertensive capability, beneficial for human health. Further work could be undertaken to gain a better understanding of the health benefits of butternut squash extracts including antibacterial, anti‐inflammation, and antitumor.

## CONFLICT OF INTEREST

The authors declare that there is no conflict of interest.
